# Immunization Coverage and Its Determinants Among Children Aged 12–23 Months in East Africa: A Bayesian Hierarchical Modeling Approach Based on DHS 2019–2022

**DOI:** 10.1155/bmri/6645541

**Published:** 2025-10-03

**Authors:** Simachew Getaneh Endalamew, Ejigu Gebeye, Solomon Keflie Assefa, Bisrat Misganaw Geremew

**Affiliations:** ^1^ Department of Veterinary Epidemiology and Public Health, School of Veterinary Medicine, Bahir Dar University, Bahir Dar, Ethiopia, bdu.edu.et; ^2^ Department of Biostatistics and Epidemiology, Institute of Public Health, College of Medicine and Health Science, University of Gondar, Gondar, Ethiopia, uog.edu.et

**Keywords:** Bayesian approach, children, determinants, East Africa, vaccination coverage

## Abstract

**Background:**

Vaccines constitute a fundamental component of public health interventions, preventing the transmission of numerous diseases. Nevertheless, vaccines remain underutilized in various regions globally, particularly in East African nations, where high mortality rates among children under 5 years of age are predominantly attributable to vaccine‐preventable diseases. Consequently, this investigation is aimed at evaluating children′s vaccination coverage and its associated determinants in East Africa utilizing Bayesian hierarchical modeling based on Demographic and Health Survey (DHS) data (2019–2022).

**Methods:**

This study analyzed nationally representative data from the DHS, a standardized cross‐sectional household survey program that collects health and population data using stratified two‐stage cluster sampling. Data were drawn from surveys conducted between 2019 and 2022 in five East African countries: Ethiopia (2019), Kenya (2022), Mozambique (2022), Rwanda (2019/20), and Tanzania (2022). A Bayesian hierarchical regression model was applied to identify factors influencing vaccination coverage, evaluating four candidate models using leave‐one‐out cross‐validation to select the best fit. Statistical significance was assessed using 95% posterior credible intervals (CrIs) after confirming model convergence.

**Results:**

A total of 15,703 weighted children aged 12–23 months were included, with an overall survey response rate of 97.2%. A substantial proportion of children (73.81%) were only partially immunized, highlighting a critical gap in achieving full vaccination coverage. The Bayesian hierarchical ordinal logistic regression showed that several factors were significantly associated with the odds of being in a higher vaccination category versus a lower one. Children residing in Kenya had 3.10 times higher odds of being in a higher vaccination category compared with those in Ethiopia (AOR = 3.10; 95% CrI: 2.49–3.86). Maternal media exposure (AOR = 1.25, 95% CrI: 1.15, 1.38), maternal education (secondary or above) (AOR = 1.42, 95% CrI: 1.21, 1.67), health facility delivery (AOR = 1.53, 95% CrI: 1.19, 1.96), postnatal care visit (AOR = 1.28, 95% CrI: 1.15, 1.43), skilled birth attendance (AOR = 1.61, 95% CrI: 1.24, 2.079), and antenatal care (ANC) visits (four and above) (AOR = 4.08, 95% CrI: 3.44, 4.84) were all positively associated with higher odds of being in a higher vaccination category.

**Conclusions:**

Vaccination coverage remains low across East Africa, with significant regional disparities. These results highlight the need for focused interventions in high‐risk areas and addressing key determinants to improve childhood vaccination rates.

## 1. Introduction

Vaccination is a global health and development success story, saving millions of lives every year. One of the biggest contributions to global child health in the 20th century was the discovery of vaccines, which have significantly decreased childhood illness and mortality [1, 2]. Therefore, vaccines are the cornerstone of public health interventions, preventing the spread of many diseases and saving many lives. They are effective in the prevention of infectious children′s illnesses like measles, diphtheria–tetanus–pertussis (DTP), meningitis, and tuberculosis [3].

According to a World Health Organization (WHO) 2021 report, approximately 25.0 million (19%) children were not immunized with the basic vaccines, which represents an increase of 2.1 million from 2020 and 5.9 million from 2019. In 2022, a total of 14.3 million children did not receive basic vaccines, indicating disparities in access to immunization and other healthcare services. Additionally, another 6.2 million received only partial vaccination. Out of the 20.5 million affected children, approximately 60% reside in just 10 countries, including Angola, Brazil, the Democratic Republic of the Congo, Ethiopia, India, Indonesia, Mozambique, Nigeria, Pakistan, and the Philippines [4].

According to WHO, the African region accounted for 43% of the world′s 19.4 million unimmunized and under‐immunized children, despite ongoing efforts to improve coverage. Compared to the global immunization coverage rate of 86%, the region had the lowest coverage at 76% [5]. According to WHO estimates, one in five children lack access to life‐saving vaccines in sub‐Saharan Africa (SSA), including East African countries [6].

In recent years, Africa, particularly East Africa, has experienced a significant increase in outbreaks of vaccine‐preventable diseases (VPDs) [7]. According to the WHO 2022 report, approximately 17,500 cases were reported across the continent, representing a fivefold increase compared to 2021. Moreover, 20 African countries experienced measles outbreaks during the first quarter of 2022, which is eight more than the number reported during the first 3 months of 2021 [8]. These alarming trends emphasize the persistent underutilization of vaccines in the region, despite their proven effectiveness in preventing childhood morbidity and mortality. Consequently, high rates of death among children under five are largely attributable to VPDs such as rotavirus, DTP, measles, polio, and *Haemophilus influenzae* type b (Hib) [9]. The recent surge in outbreaks highlights the urgent need to understand and address the factors contributing to suboptimal vaccination coverage, as inadequate immunization not only increases vulnerability to VPDs but also undermines broader public health efforts to reduce child mortality.

The fundamental component of the Sustainable Development Goals (SDGs) for 2030 is ensuring equitable access to vaccines across all nations to eliminate preventable mortality among children under 5 years of age [10]. This goal aims to reduce under‐five mortality to 25 per 1000 live births and eliminate preventable neonatal and under‐five deaths by 2030 [11]. Therefore, evaluating the current vaccination coverage and its determinants is essential for achieving these goals. Identifying countries and populations with low coverage facilitates targeted interventions, ensuring resources are allocated optimally to improve immunization rates and reduce preventable child mortality.

While extensive research has focused on national‐level assessments of vaccination coverage across multiple countries [12–19], regional disparities in East Africa remain underexplored. This gap limits the availability of localized evidence needed to inform targeted policymaking. Furthermore, prior studies analyzing Demographic and Health Survey (DHS) data to evaluate immunization rates often overlook the dataset′s inherent hierarchical structure [20, 21]. Such nested data, where individual responses are grouped within communities, regions, or countries, necessitate multilevel regression modeling. Single‐level analytical approaches risk producing biased parameter estimates and misleading standard errors due to unaccounted clustering effects. To address these limitations, this study employs Bayesian hierarchical ordinal regression models to investigate regional inequities in child immunization coverage using recent DHS datasets from five East African nations.

This research provides up‐to‐date insights into vaccination coverage and the risk factors influencing vaccine uptake, contributing to the existing body of knowledge on childhood immunization. The findings can serve as a valuable resource for policymakers and nongovernmental organizations striving to reduce child mortality by designing and implementing targeted interventions tailored to the specific needs of children aged 12–23 months in East African countries. Accordingly, the primary objective of this study was to assess childhood vaccination coverage and its determinants across East Africa using a hierarchical ordinal regression model, applying a Bayesian approach.

## 2. Methods and Materials

### 2.1. Study Setting and Population

This study was conducted in East African countries and utilizes a community‐based cross‐sectional survey design called DHS. The survey was conducted from 2019 to 2022. The specific East African countries with their survey years included in this study were Ethiopia (2019), Kenya (2022), Mozambique (2022), Rwanda (2019/20), and Tanzania (2022). The most recent standard DHS report available for these countries was obtained and used as the data source for this study.

The target population for this study was all children aged 12–23 months in East African countries. The study population included children aged 12–23 months, whose parents provided information about vaccination status during data collection. Children were included in this study if their vaccination history was reported by their mother or verified through a vaccination card.

However, children in this age group who were no longer alive at the time of the survey or had missing or uncertain vaccination data (e.g., responses marked as “do not know”) were excluded. Additionally, to ensure the data accurately reflects current vaccination coverage, we only included countries that conducted surveys from 2019 onward and followed the DHS‐8 survey guidelines, which incorporate updated measurements on child and maternal health [22].

### 2.2. Sampling Methods, Data Collection Procedure, and Quality Control

**Figure 1:**
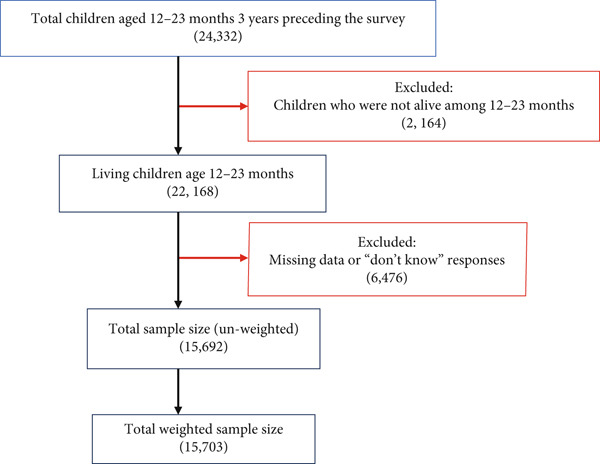
Schematic representation of sampling procedure of vaccination status by children in East African countries.

The DHS employs a stratified, two‐stage cluster sampling design for each country. Stratification is based on geographic regions and is further divided into urban and rural areas within each region. In the first stage, enumeration areas (also known as primary sampling units (PSUs)) are selected using probability proportional to size (PPS) within each stratum. In the second stage, a complete household listing is conducted within the selected clusters. From this listing, a fixed number of households is then chosen through equal probability systematic sampling (EPSS). To account for unequal selection probabilities at the cluster level and adjust for nonresponse, sampling weights are applied. These weights help minimize selection bias and ensure representative estimates. The DHS follows standardized sampling procedures across all countries, ensuring consistency in survey methodology. A schematic representation of the sampling design is provided below (Figure 1).

The DHS collected its core data through face‐to‐face interviews with women aged 15–49. These interviews focused on health‐related details and immunization histories of their children, specifically those aged 12–23 months and 24–35 months. The survey instrument included variables including socio‐demographic factors (e.g., education and household wealth), socioeconomic conditions, and maternal health indicators such as pregnancy outcomes and antenatal care. During data collection, mothers were asked to present vaccination records for their children or, if unavailable, to verbally confirm whether their children had received recommended vaccines. Responses indicating “no vaccinations” were recorded as children having no vaccine coverage.

To ensure data quality, the DHS employed structured, standardized questionnaires that were pretested for clarity and reliability. Rigorous protocols were followed, including training field staff (enumerators, supervisors, and editors) to minimize errors; conducting real‐time monitoring during fieldwork; translating questionnaires into local languages to improve participant understanding; and collaborating with data processing experts to manage and clean the dataset. Additional steps were taken to reduce systematic biases, such as selection bias or recall bias, during both data collection and analysis. For more details on the DHS data collection methodology, refer to the official DHS documentation [22].

### 2.3. Study Variables

The primary outcome of interest was the immunization status of children aged 12–23 months. In line with the DHS‐8 guideline and national immunization schedules [22, 23], this included vaccines such as Bacillus Chalmette–Guerin (BCG), DPT‐HepB‐Hib (PENTA), pneumococcal conjugate vaccine (PCV), oral polio vaccine (OPV), rotavirus vaccine, inactivated polio vaccine (IPV), and measles‐containing vaccine (MCV). Children were grouped into three categories: fully immunized, partially immunized, and nonimmunized (zero‐dose). Formally, the outcome variable *Y*_*i* *k*_, for the *i*th child in the *k*th cluster was coded as follows:

Yik=0:if the child is not immunized,1:if the child is partially immunized,2:if the child is fully immunized.

The independent variable selection process was based on different literature [24–28] and the availability of data at individual and community levels. A hierarchical approach was used, with two levels of explanatory variables.

Individual‐level variables include indicators of the child and mother′s sociodemographic and obstetrical characteristics, such as the mother′s age, educational level, presence of a skilled birth attendant during delivery, place of delivery, exposure to media, birth order, wealth status, antenatal care follow‐up, postnatal care follow‐up, sex of the child, and sex of household head. Community‐level variables include the place of living country, residence, and distance to health facility.

### 2.4. Operational Definitions for the Outcome Variables

*Fully vaccinated*: As per the DHS‐8 guideline [22] and national immunization schedules [23], a child aged 12–23 months is considered fully vaccinated if they have received all vaccines recommended for their age by the time of the survey.

*Incomplete vaccination:* This is defined as a child missing at least one dose of any of the vaccines given to children aged 12–23 months of age.

*Truly zero-dose children:* Children 12–23 months who have not received any doses of each of the above vaccines, per DHS‐8 data [23, 29].

### 2.5. Data Management and Statistical Analysis Methods

Data cleaning and preprocessing were conducted in R software [30]. Missing data were examined for patterns, and variables missing by design (e.g., husband′s education for single mothers) were excluded from multivariable models to avoid bias. For variables with < 5% missingness, Little′s MCAR test indicated that data were missing at random; thus, multiple imputation via chained equations (MICE) with logistic regression was applied to generate 10 imputed datasets. Analyses were weighted to account for the sampling design, and the results are reported as weighted estimates.

Descriptive statistics were used to summarize the population characteristics, and bivariable analyses were used to identify potential covariates. Factors associated with vaccination coverage were examined using a Bayesian hierarchical ordinal logistic regression model fitted with the brms package in R [31], employing Hamiltonian Monte Carlo with No‐U‐Turn Sampler (NUTS) [32]. Four models (null, individual‐level, community‐level, and full models) were compared using leave‐one‐out cross‐validation (LOO‐CV).

Model convergence and fit were assessed using standard Bayesian diagnostics (Gelman–Rubin statistic [33], effective sample size, *R*‐hat values, trace plots, and posterior predictive checks). Clustering measures (ICC [34], median odds ratio [MOR] [35], and PCV [36, 37]) were computed to assess variability across enumeration areas. Further details on the Bayesian hierarchical ordinal regression model, prior specification, measures of cluster heterogeneity, and diagnostic thresholds are provided in File S1.

### 2.6. Ethical Consideration and Consent

This study utilized a publicly available secondary dataset from the Measure DHS Program. As a result, ethical approval and individual participant consent were not required. However, formal permission was obtained from the DHS program data archivists to access and use the data for this research. The dataset remained confidential, with all personally identifiable information removed. Furthermore, the data were strictly used for this authorized study and were not shared with other researchers.

## 3. Results

### 3.1. Sociodemographic, Economic, and Obstetrical Characteristics of Respondents

This study included reproductive‐age women aged 15–24 years with a weighted sample size of 15,703 in East Africa. The number of children aged between 12 and 23 months included in this study varied across the East African countries. Tanzania had the highest number of participants, with 4274 (27.22%) children. In contrast, Ethiopia had the lowest number of participants, with only 1015 (6.46%) children (Table 1).

**Table 1:** Description of surveys and sample size characteristics of East African countries.

**Countries**	**Survey year**	**Sample size (** **n** **)**	
		**Unweighted frequency (%)**	**Weighted frequency (%)**
Ethiopia	2019	1002 (6.39)	1015 (6.46)
Kenya	2022	3918 (24.97)	3623 (23.07)
Mozambique	2022/23	3411 (21.74)	3544 (22.57)
Rwanda	2019/20	3139 (20.00)	3247 (20.68)
Tanzania	2022	4222 (26.91)	4274 (27.22)

Among the study cohort, almost half (50.20%) of mothers attained a primary education. The majority (91.08%) of the women had ANC visits for their recent pregnancy. Of those who had ANC visits, 57.80% had four or more times of visits. However, only 19.94% of mothers received a postnatal care checkup within 2 months after delivery. Regarding delivery sites, the majority (80%) of mothers were delivered at health facilities. The majority 11,185 (71.23%) of respondents were from rural areas. Regarding marital status, the majority 12,957 (82.51%) of participants were cohabiting with their partners at the time of the study (Table 2).

**Table 2:** Sociodemographic and obstetric characteristics of children with their vaccination status in East African countries, from their recent DHS.

**Variables**	**Categories**	**Unweighted frequency (%)**	**Weighted frequency (%)**
Residence	Rural	11,158 (71.11)	11,185 (71.23)
	Urban	4534 (28.89)	4517 (28.77)

Maternal education	No education	3374 (21.50)	3021 (19.24)
	Primary education	7453 (47.50)	7882 (50.19)
	Secondary or higher	4865 (31.00)	4800 (30.57)

Wealth status	Poor	6845 (43.62)	6722 (42.81)
	Middle	2957 (18.84)	2908 (18.52)
	Rich	5890 (37.54)	6073 (38.67)

Marital status	Not living together	2654 (16.91)	2746 (17.49)
	Living together	13,038 (83.09)	12,957 (82.51)

Birth order	First	4028 (25.67)	4102 (26.12)
	Second to third	9212 (58.71)	9361 (59.62)
	Fourth and above	2452 (15.62)	2240 (14.26)

Distance to healthcare	Big problem	5091 (32.44)	5064 (32.25)
	Not a big problem	10,601 (67.56)	10,639 (67.75)

Maternal age	15–24	5190 (33.07)	5342 (34.02)
	25–34	7088 (45.17)	7048 (44.88)
	35–49	3414 (21.76)	3313 (21.10)

Media exposure	No media	7605 (48.46)	7280 (46.36)
	Have media	8087 (51.54)	8423 (53.64)

Skilled birth attendant	Not skilled	2805 (17.88)	2722 (17.34)
	Skilled	12,887 (82.12)	12,980 (82.66)

Child sex	Male	7982 (50.87)	7959 (50.69)
	Female	7710 (49.13)	7744 (49.31)

Delivery site	Home	3225 (20.56)	3141 (20.00)
	Health facility	12,466 (79.44)	12,562 (80.00)

Postnatal care visit	No	12,655 (80.65)	12,572 (80.06)
	Yes	3037 (19.35)	3131 (19.94)

Visited by HEWS	No	13,358 (85.13)	13,527 (86.14)
	Yes	2334 (14.87)	2176 (13.86)

Antenatal care visit	No visit	1321 (8.42)	1401 (8.92)
	1–3	5223 (33.28)	5226 (33.28)
	≥ 4	9148 (58.30)	9076 (57.80)

Abbreviations: ANC, antenatal care; HEWs, health extension workers.

#### 3.1.1. Children Immunization Coverage

**Figure 2:**
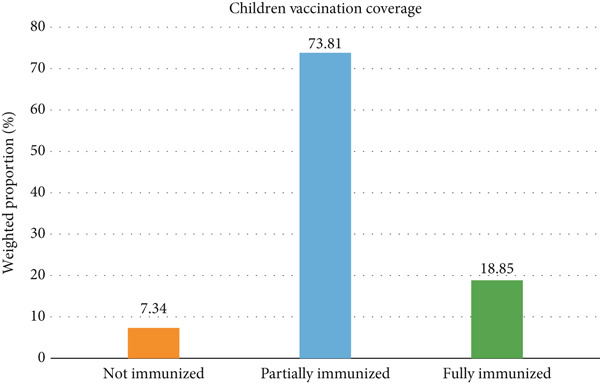
Immunization coverage of children aged 12–23 months in East Africa.

The study analyzed vaccination coverage among 15,703 children aged 12–35 months across selected East African countries (Ethiopia, Kenya, Tanzania, Rwanda, and Mozambique), with immunization status verified through health cards or caregiver recall. The majority of children (73.81%, 95% CI: 72.67%–74.95%) were partially immunized, failing to complete the full course of age‐appropriate vaccines, while 7.34% (95% CI: 6.47%–8.20%) of children received no vaccines from the recommended national schedules (Figure 2).

### 3.2. Determinants of Childhood Vaccination Coverage in East Africa

#### 3.2.1. Measures of Variation (Random Effects)

Initially, in the null model, significant variations in the odds of children′s vaccination coverage were attributed to cluster differences (*σ* ^2^ = 2.06). This model accounted for 39% of the overall variability in vaccination status difference due to disparities between clusters (ICC = 0.39). Subsequent models exhibited fluctuations in the between‐cluster variances. Model II, focusing solely on individual‐level factors, displayed a slightly reduced between‐cluster variance (ICC = 0.24). Notably, the transition from Model I to Model II showed a decrease in ICC. Model IV, incorporating both individual‐ and community‐level variables, saw the ICC decrease (ICC = 0.14), signifying that differences in cluster characteristics substantially contribute to variations in vaccination status. The marked reduction in ICC from the null to the fully adjusted model highlights the dominant role of individual‐level determinants, such as maternal education, in driving disparities in vaccination coverage. This suggests that policies aimed at improving maternal education, enhancing household health awareness, and addressing household‐level barriers could significantly reduce geographic inequalities in vaccination uptake. The combined influence of individual‐ and community‐level factors explained approximately 73% (proportional change in variance = 0.73) of the variation in vaccination status observed between clusters (Table 3).

**Table 3:** Measure of variation and model fitness.

	**Null model**	**Model II**	**Model III**	**Model IV**
Random effect result				
Variance	2.06	1.03	0.99	0.55
ICC (%)	0.39	0.24	0.23	0.14
PCV (%)	Ref	0.5	0.52	0.73
MOR (%)	3.93	2.62	2.58	2.03
Model fit statistics				
LOO‐CV	19,034.6	19030.7	19,028.5	19,022.3 ^∗^

*The selected model value.

Moreover, the MOR confirmed that community‐level factors significantly influenced the odds of vaccination status. The disparity in vaccination coverage odds between communities, which was unexplained in the null model, was reduced by a factor of 1.9 in the final model, which included both individual‐ and community‐level factors. This indicates that when a child moves from a high‐risk to a low‐risk neighborhood, the risk of not being vaccinated decreases by 90%.

Based on the LOO‐CV criterion, the Bayesian multilevel ordinal logistic regression model (Model IV), with both community‐level and individual‐level variables, was found to be the best model to fit the data with minimum LOO‐CV values of 19022.3 (Table 3 and Figure S1).

#### 3.2.2. Measures of Association (Fixed Effects)

The posterior mean and 95% credible intervals for the fixed effects of the four models are shown in Table 4. The outcome of the Bayesian hierarchical ordinal model using the cumulative distribution revealed that variables, including country, maternal age, maternal education, PNC, skilled birth attendance, place of delivery, media exposure, wealth index, birth order, and ANC visits, were statistically significant based on their credible interval results. The sign of the covariate coefficient indicates whether a variable is associated with higher or lower levels on the vaccination coverage category rating scale compared with the baseline (Table 4).

**Table 4:** Posterior summary estimates (mean) of the selected model.

**Parameter**	**Estimate (** **β** **coefficients)**	**Estimate error**	**AOR (95% CrI)**
**θ**_1_	0.45 (0.18, 0.74)	0.14	1.57 (1.20, 2.09)
**θ**_2_	0.87 (0.81, 0.96)	0.16	2.39 (2.25, 2.61)
Country (ref = Ethiopia)			
Kenya	1.13 (0.91, 1.35)	0.11	3.10 (2.49, 3.86) ^∗^
Mozambique	0.76 (0.54, 0.98)	0.11	2.14 (1.72, 2.67) ^∗^
Rwanda	2.70 (2.47, 2.94)	0.12	14.91 (11.76, 18.95) ^∗^
Tanzania	0.67 (0.45, 0.90)	0.11	1.96 (1.57, 2.45) ^∗^
Maternal age (ref = 15–24)			
25–34	0.38 (0.33, 0.64)	0.03	1.46 (1.39, 1.90) ^∗^
35–45	0.45 (0.42, 0.67)	0.01	1.57 (1.52, 1.95) ^∗^
Residence (ref = rural)			
Urban	−0.06 (−0.18, 0.07)	0.06	0.94 (0.83, 1.07)
Media exposure (ref = no)			
Yes	0.23 (0.14, 0.32)	0.05	1.26 (1.15, 1.38) ^∗^
Wealth index (ref = poor)			
Middle	0.11(‐0.00, 0.23)	0.06	1.12 (0.99, 1.26)
Rich	0.15 (0.03, 0.28)	0.07	1.17 (1.03, 1.33) ^∗^
Antenatal care visit (ref = no ANC visit)			
One to three	1.19 (1.02, 1.36)	0.09	3.28 (2.77, 3.88) ^∗^
Four and above	1.41 (1.24, 1.58)	0.09	4.08 (3.44, 4.85) ^∗^
Maternal education (ref = no education)			
Primary education	0.35 (0.19, 0.51)	0.06	1.41 (1.29, 1.66) ^∗^
Secondary or above	0.38 (0.25, 0.51)	0.08	1.46 (1.21, 1.67) ^∗^
Delivery site (ref = home)			
Health facility	0.42 (0.17, 0.67)	0.13	1.53 (1.19, 1.96) ^∗^
Postnatal care visit (ref = no)			
Yes	0.25 (0.14, 0.35)	0.05	1.28 (1.15, 1.43) ^∗^
Skilled birth attendance (ref = no)			
Yes	0.23 (0.14, 0.32)	0.13	1.61 (1.24, 2.08) ^∗^
Marital status (ref = not living together)			
Living together	0.02 (−0.10, 0.15)	0.06	1.02 (0.90, 1.16)
Child sex (ref = male)			
Female	−0.07 (−0.17, 0.04)	0.04	0.95 (0.88, 1.03)
Distance to health facility (ref = big problem)			
Not a big problem	0.01 (−0.09, 0.10)	0.05	1.01 (0.92, 1.11)
Visited by HEWS (ref = no)			
Yes	−0.26 (−0.38, 0.14)	0.06	0.77 (0.69, 1.15)
Parity (ref = first order)			
Second to third order	−0.19 (−0.30, −0.08)	0.06	0.83 (0.74, 0.93) ^∗^
Fourth and above order	−0.45 (−0.63, −0.28)	0.09	0.63 (0.53, 0.76) ^∗^

Abbreviations: AOR, adjusted odds ratio; CrI, posterior credible interval; ref = reference.

*Statistically significant variables.

The positive country‐specific coefficients indicate that residing in Kenya, Mozambique, Rwanda, or Tanzania had higher odds of being in a higher vaccination category versus a lower one (e.g., fully vs. partially) compared to those in Ethiopia. After controlling for other covariates and accounting for cluster‐level variability, the odds of being in a higher vaccination category were approximately 3.10 (AOR = 3.10; 95% CrI: 2.49–3.86) times greater for children in Kenya, 2.14 (AOR = 2.14; 95% CrI: 1.72–2.67) times greater in Mozambique, 14.91 (AOR = 14.91; 95% CrI: 11.76–18.95) times greater in Rwanda, and 1.96 (AOR = 1.96; 95% CrI: 1.57–2.45) times greater in Tanzania relative to Ethiopia.

Several maternal, household, and healthcare factors were strongly associated with the odds of a child being in a higher vaccination category versus a lower one (e.g., fully vs. partially). Children born to women aged 25–34 years had 1.46 times the odds of being in a higher vaccination category versus a lower one (e.g., fully vs. partially) (AOR = 1.46; 95% CrI: 1.33–1.64), and those whose mothers were 35–45 years old had 1.57 times higher odds of being in a higher vaccination category versus a lower one (e.g., fully vs. partially) (AOR = 1.57; 95% CrI: 1.42–1.67), as compared with children born to women aged 15–24 years. Media exposure was also strongly associated, with children born to women with access to media being 1.26 times the odds of being in a higher vaccination category versus a lower one (AOR = 1.26; 95% CrI: 1.15–1.38) than those without such access. Similarly, children from the wealthiest households were 1.17 times the odds of being in a higher vaccination category versus a lower one (AOR = 1.17; 95% CrI: 1.08–1.27) than those from the poorest households.

Maternal healthcare utilization emerged as a strong determinant of children′s vaccination outcomes. Compared with children born to women with no ANC visits, those whose mothers attended one to three visits had 3.28 times the odds of being in a higher vaccination category versus a lower one (AOR = 3.28; 95% CrI: 2.77–3.89), while attendance at four or more visits was associated with a 4.08‐fold increase (AOR = 4.08; 95% CrI: 3.46–4.82). Maternal education showed a similar pattern: children born to women with primary education had 1.41 times the odds of being in a higher vaccination category (AOR = 1.41; 95% CrI: 1.21–1.61), and those whose mothers had secondary or higher education had 1.46 times the odds of being in a higher vaccination category (AOR = 1.46; 95% CrI: 1.28–1.66) compared with children born to women with no formal schooling.

Delivery and postnatal care utilization also influenced vaccination outcomes. Birth in a health facility increased the odds of being in a higher vaccination category versus a lower one by 1.52 times (AOR = 1.52; 95% CrI: 1.19–1.96) compared with those delivered at home. Similarly, skilled birth attendance was associated with 1.26 times greater odds of being in a higher vaccination category relative to unassisted births (AOR = 1.26; 95% CrI: 1.15–1.38). Postnatal care attendance further increased the odds of being in a higher vaccination category by 1.60 times compared with no PNC visits (AOR = 1.60; 95% CrI: 1.25–2.08).

Finally, birth order showed an inverse association with vaccination status. Compared with first‐born children, those of second or third birth order had 0.83 times the odds of being in a higher vaccination category versus a lower one (AOR = 0.83; 95% CrI: 0.74–0.92), while children of fourth or higher birth order had 0.64 times the odds (AOR = 0.64; 95% CrI: 0.53–0.74).

## 4. Discussion

This study utilized a hierarchical ordinal regression model to analyze childhood vaccination coverage, enabling the identification of children at higher risk of being unvaccinated and highlighting priority areas for intervention. This study employed various statistical models to analyze childhood vaccination coverage, including categorical (multinomial) models, proportional odds (PO) or cumulative models, adjacent‐categories logit models, and sequential logit models. Model fit was assessed using LOO‐CV values. Based on this criterion, the cumulative logit model was identified as providing the best fit for determining the factors influencing immunization coverage.

A concerning 7% (95% CI: 6.47%–8.20%) of children received no vaccines from the recommended national schedules. This proportion exceeds the WHO regional estimate of 5% for zero‐dose children in SSA. This higher proportion likely reflects disparities in fragile and conflict‐affected areas within the region [38]. Meanwhile, the majority of children (74%, 95% CI: 72.67%–74.95%) were partially immunized, having started but not completed the full course of age‐appropriate vaccines. This aligns with regional dropout rates, which range between 18% and 22% from initial vaccines such as BCG to later doses like the measles vaccine in East Africa. Only 18.85% (95% CI: 17.78%–19.95%) of children achieved full immunization, falling well below the Global Vaccine Action Plan (GVAP) target of 90% coverage for basic vaccines coverage by 2020 [39, 40].

Zero‐dose and partially immunized children represent two distinct public health challenges that require tailored interventions. Zero‐dose children often face barriers such as limited healthcare access, socioeconomic disadvantage, and lack of awareness that prevent vaccination [41, 42]. Addressing this group requires targeted outreach and community engagement to improve initial contact with immunization services [43].

In contrast, partially immunized children face different obstacles, including missed opportunities during health visits, weak follow‐up systems, and inconsistent vaccine supply [44]. Strengthening routine immunization programs, improving follow‐up mechanisms, and enhancing caregiver education are critical to ensure the completion of the full vaccine schedule [45]. Recognizing these distinct challenges is essential for designing effective strategies to improve overall vaccination coverage across East Africa.

The study identified several significant predictors of children′s immunization status in the study area, including country of residence, maternal age, mother′s education, antenatal care visits, postnatal care visits, delivery site, skilled birth attendance, birth order, and media exposure.

The study showed that country of residence emerged as a key determinant of childhood immunization outcomes. Children residing in Kenya, Tanzania, Rwanda, and Mozambique were substantially more likely to achieve complete or partial immunization compared with those in Ethiopia. These cross‐country disparities likely reflect differences in national health policies, healthcare infrastructure, and resource allocation. Variations in immunization coverage may be attributed to factors such as the prioritization of childhood vaccination within national health agendas, the strength of vaccine supply chains, and the effectiveness of community‐based outreach programs. Countries with well‐integrated immunization strategies [46], greater accessibility to health facilities [47, 48], and robust public health communication systems tend to achieve higher coverage rates [49]. Conversely, systemic challenges, including resource limitations, geographic barriers, and logistical constraints in vaccine distribution, may contribute to suboptimal coverage in other settings [50, 51].

Maternal education significantly impacts childhood vaccination status. Children born to educated mothers were more likely to be in higher vaccination categories than those born to noneducated parents. This association may be attributed to the role of education in improving health literacy, promoting positive health‐seeking behaviors, and enhancing the ability to navigate healthcare systems. Educated mothers are more likely to understand the benefits of immunization, adhere to vaccination schedules, and utilize available health services, which collectively contribute to higher immunization uptake. These findings align with previous studies demonstrating that maternal education is consistently associated with improved child health outcomes, including higher rates of timely and complete vaccination [52, 53].

An increase in maternal age was found to be a significant factor that affects children′s vaccination status. Older mothers were more likely to have children in higher vaccination categories, consistent with previous studies indicating a positive association between maternal age and full immunization coverage [54, 55]. Household wealth also emerged as a key predictor: children from wealthier families had higher odds of being in higher vaccination categories compared with those from economically disadvantaged households [55, 56]. This finding aligns with numerous studies that demonstrate a positive correlation between household economic status and the likelihood of complete immunization. Even though vaccination services are typically provided free of charge, indirect costs such as transportation, lost wages, and opportunity costs may pose barriers to access [44, 57]. These additional financial burdens appear to disproportionately affect the most economically disadvantaged families.

The study found that access to media is a significant predictor of childhood immunization status, with children of mothers with access to media being more likely to be in higher vaccination categories compared with those whose mothers had no media exposure. This is likely because the media serves as an important channel for disseminating health information, increasing awareness about the benefits of vaccinations, providing reminders, and correcting misinformation, all of which contribute to higher vaccination rates [58–60]. These findings were in line with previous literature that showed having media exposure correlates with access to health‐related information, which can further contribute to an increased awareness of parents for child health through vaccination [19].

Antenatal care visits during pregnancy and postnatal care visits after birth are significant predictors of childhood immunization coverage. Children whose mothers attended both ANC and PNC were more likely to be in higher vaccination categories compared to those whose mothers did not utilize these care visits. This finding aligns with previous research [61], which highlights that both antenatal and postnatal care play crucial roles in ensuring higher rates of childhood vaccination. Health institutions contribute to improved immunization outcomes by providing immediate vaccination services, enhancing parental education [62], ensuring structured follow‐up care, integrating vaccinations into comprehensive care plans [63], reducing access barriers [64], and enabling early detection and management of health issues.

The study also found that children born at health institutions are more likely to be in higher vaccination categories compared to those born at home. This might be because health facilities provide immediate vaccination services, offer educational programs that increase parental awareness, ensure structured follow‐up care, integrate vaccinations into antenatal and postnatal care, reduce barriers to accessing healthcare, and enable early detection of health issues. These factors collectively contribute to higher immunization rates, as supported by existing research [65, 66].

Similarly, skilled birth attendance was positively associated with childhood vaccination uptake. Children delivered by skilled personnel were more likely to be in higher vaccination categories. Different literatures have found that children born in health facilities are significantly more likely to complete essential vaccinations such as measles, polio, and DTP than those born at home or in informal settings [67, 68]. This might be due to skilled birth attendance fostering early and continuous contact with the healthcare system for follow‐up visits; enhancing maternal education and awareness about immunization, building trust in medical interventions, which reduces vaccine hesitancy; and ensuring better record keeping and monitoring of vaccination schedules [69–71].

Later‐born children are less likely to be in higher vaccination categories compared to first‐born children, highlighting a potential birth‐order effect on vaccination status. Aligned with the present findings, earlier studies revealed that first‐born children tended to have better immunization indicators than children with later birth orders [72, 73]. This may be attributed to several factors, including increased parental burden, resource dilution, and reduced healthcare utilization in larger families [74]. Parents with multiple children may face logistical challenges, time constraints, and competing caregiving responsibilities, leading to delays or missed vaccinations [75]. Additionally, maternal health‐seeking behavior and engagement with immunization services may decline with successive births, particularly in resource‐limited settings where access to healthcare and health education is constrained [72, 76].

### 4.1. Strengths and Limitations of the Study

The research utilized nationally representative DHS data, providing a comprehensive perspective on vaccination status across multiple nations. This extensive coverage enhances the study′s validity and generalizability. Furthermore, the implementation of a Bayesian hierarchical ordinal model facilitates the examination of childhood vaccination variations, effectively accounting for both observable and unobservable factors.

Nevertheless, the study faced certain constraints. The DHS data were collected from surveys conducted up to 3 years prior for assessing vaccination coverage, relying on participants′ ability to accurately remember past events and present their children′s vaccination cards. Mothers′ verbal reports for vaccination status may introduce maternal recall bias, potentially leading to an overestimation of vaccination coverage if caregivers overreport immunization status, or an underestimation if some doses were missed during recall. Additionally, since the information was gathered through the women′s questionnaire, it only included children with living biological mothers at the time of the survey, introducing selection bias. This may have skewed coverage estimates if children without surviving mothers differed systematically in vaccination status. Finally, the model did not incorporate all possible variables like maternal occupation, maternal toxoid vaccination status, and knowledge and awareness influencing childhood vaccination coverage. The omission of these factors may have limited the comprehensiveness of the analysis and could have affected the magnitude or direction of associations observed.

## 5. Conclusion and Recommendations

Based on these findings, there were significant variations in the vaccination status of children across East Africa. The disparities among countries reflect underlying differences in healthcare access, quality, and socioeconomic conditions. Several factors were identified as influencing childhood immunization uptake among children aged 12–23 months, including maternal age, marital status, educational level, media exposure, postnatal care, parity, and antenatal care visits, all of which were significant predictors of vaccination coverage in East Africa.

Given the substantial influence maternal factors exert on their children′s vaccination status, addressing maternal concerns and barriers is essential when developing public health interventions aimed at promoting childhood immunizations. Expanding healthcare infrastructure in underserved rural areas through mobile clinics and community outreach is essential for improving vaccine accessibility. Encouraging pregnant women to attend at least four antenatal care visits and incorporating vaccination counseling and scheduling into each visit plays a critical role in increasing childhood immunization coverage. Including vaccine education in school curricula helps to foster awareness and support among young people. Strengthening funding and partnerships to deliver culturally sensitive vaccination campaigns can help reach marginalized populations. Tailoring interventions to the unique needs of communities with equitable resource distribution enhances their effectiveness. Additionally, leveraging further research and geospatial tools to identify local barriers allows precise, targeted efforts to raise vaccination rates across East Africa.

## Consent

This study utilized existing data from the EDHS, and therefore, participant consent was not required. A written letter of permission was obtained from the DHS program data archivists to download and use the data for this study. The survey data were obtained from the USAID‐DHS program, and the researchers ensured strict confidentiality throughout the study.

## Conflicts of Interest

The authors declare no conflicts of interest.

## Author Contributions

Conceptualization: Simachew Getaneh Endalamew. Data curation and software and formal analysis: Simachew Getaneh Endalamew and Solomon Keflie Assefa. Methodology: Simachew Getaneh Endalamew. Supervision and validation: Bisrat Misganaw Geremew and Ejigu Gebeye. Writing—original draft: Simachew Getaneh Endalamew. Writing—review and editing: Simachew Getaneh Endalamew, Bisrat Misganaw Geremew, Solomon Keflie Assefa, and Ejigu Gebeye.

## Funding

No funding was received for this manuscript.

## Supporting information


**Supporting Information** Additional supporting information can be found online in the Supporting Information section. File S1: Additional details on the statistical methods used in this study. It outlines the Bayesian hierarchical ordinal logistic regression framework, assessment of clustering and heterogeneity, specification of priors and distributions, and model diagnostics. Figure S1 and S2: Model comparison and convergence checks to demonstrate the robustness of the results, respectively.

## Data Availability

Data utilized for this research was obtained from EDHS accessed through the website (https://www.dhsprogram.com), and the dataset used for the final analysis during the current study can be obtained from the corresponding author on reasonable request.
