# Supplementary material for: Immunization Coverage and Its Determinants Among Children Aged 12–23 Months in East Africa: A Bayesian Hierarchical Modeling Approach Based on DHS 2019–2022
Source: Biomed Res Int. 2025 Oct 3;2025:6645541. doi: 10.1155/bmri/6645541 (PMC12491837; doi:10.1155/bmri/6645541)
Supplement: Supplementary file 1 — Supporting Information Additional supporting information can be found online in the Supporting Information section. File S1: Additional details on the statistical methods used in this study. It outlines the Bayesian hierarchical ordinal logistic regression framework, assessment of clustering and heterogeneity, specification of priors and distributions, and model diagnostics. Figure S1 and S2: Model comparison and convergence checks to demonstrate the robustness of the results, respectively. [file BMRI-2025-6645541-s001.docx]

## **Supplementary Document s1**

## Methodology

## Model

Bayesian hierarchical ordinal logistic regression model was employed to investigate the factors linked to vaccination coverage in East Africa. The relationship between vaccination status and various factors was analyzed using a multilevel mixed-effects ordinal logistic regression model with a Bayesian approach incorporating various baseline distributions. To capture unobserved factors that contributed to heterogeneity, a random effect model was included to the model.

The main characteristic of the Bayesian method is that it uses a probability function to measure uncertainty in statistical inference. In other words, its probability function can be used as a benchmark for a researcher’s trust in an event. Applying Bayes’ [1] rule, the posterior distribution ($p\left( \theta|y \right)$) for the model’s parameters, $\theta$, can be written as:

$$p\left( \theta|y \right)\propto p\left( \theta\right)*p\left( y|\theta\right) \left( 1 \right)$$

Where, $p\left( \theta\right)$ is the prior distribution, and $p\left( y|\theta\right)$ is the likelihood obtained from the data.

The Bayesian hierarchical ordinal regression model was fitted using the brms package in R [2], which employs Hamiltonian Monte Carlo (HMC) with the No-U-Turn Sampler (NUTS) [3]. This package allows for Bayesian multilevel modeling in R through Stan, a probabilistic programming platform that performs full Bayesian inference using HMC. The analysis was conducted with 16,000 iterations, discarding the first 1,000 as a warm-up, and utilizing four chains to ensure robust sampling. To evaluate model convergence, several diagnostic checks were performed, including posterior predictive checks to compare densities between replicated and observed data, R-hat values to confirm chain convergence, Bulk and Tail Effective Sample Size (ESS) to assess sampling adequacy, and trace plots to ensure proper chain mixing. Additionally, sensitivity analyses were conducted by testing different prior specifications to examine their influence on parameter estimates.

## Ordinal Logistic regressions

Ordinal regression is a set of statistical techniques designed to analyze ordered categorical outcomes, with each model differing in its underlying assumptions and logit structure [4]. Commonly used approaches include the baseline-category logit, proportional odds model (POM), continuation ratio model (CRM), and adjacent category model (ACM) [5].

The POM, widely applied in practice, evaluates the cumulative probability of being in a higher versus lower category. It assumes that observed outcomes are derived from a continuous latent variable that has been categorized. In contrast, CRMs assess the likelihood of progressing to a higher category relative to remaining in a lower one, making them useful for modeling stepwise transitions. ACMs, on the other hand, focus on the probabilities of moving between adjacent categories, often assuming distinct latent processes at each stage [6]. Unlike the POM, which models cumulative odds across grouped categories, CRMs estimate conditional odds based on reaching or surpassing a particular threshold. This distinction allows CRMs to offer deeper insights into sequential decision-making processes, making them particularly useful in studies where progression through categories follows a structured pathway [7, 8].

## Prior and distribution specifications

Since there is no prior information from previous studies, the regression coefficients were assumed to follow a normal distribution for all model regression coefficients$\beta$.

$$\beta\sim N\left( 0,1000 \right) \left( 2 \right)$$

For the intercept term, the student’s t-distribution was used with 3 degrees of freedom, a location parameter of 1.6, and a scale parameter of 2.5 as our prior distribution.

$$\beta_{0} \sim Student t\left( 3,1.6,2.5 \right) \left( 3 \right)$$

The choice of the student’s t-distribution allows to account for potential heavier tails in the data compared to a normal distribution, making the model more robust to outliers. The three degrees of freedom imply that the tails of the distribution are less sensitive to extreme values, providing a more flexible representation of uncertainty.

For the random effect (variance) and the shape parameter $\sigma$, a half-Cauchy prior was chosen. This choice was influenced by a recommendation that suggested the half-Cauchy prior is a preferable option for a variance parameter (59). The half-Cauchy distribution with a scale of 25 serves as an almost flat prior, though not entirely. Having a prior distribution that is not entirely flat offers sufficient information for the numerical approximation algorithm to effectively explore the target density, which is the posterior distribution.

$$\sigma\sim HC\left( 0,25 \right) \left( 4 \right)$$

## Model diagnostics and comparison

To evaluate model performance, we conducted leave-one-out cross-validation (LOO-CV) using the “LOO” package in R [9]. The optimal model was identified by achieving the lowest LOO-CV score, indicating superior predictive accuracy. Following model selection, we developed and compared four hierarchical Bayesian models to assess the association of individual- and community-level factors with vaccination status, which includes Model I (Null Model): which captured baseline variation in vaccine coverage without explanatory variables; Model II (Individual-Level Model): which incorporated only individual-level predictors (e.g., maternal education, household wealth); Model III (Community-Level Model): focused on community-level factors (e.g., regional healthcare access, urban/rural residence) and Model IV (Full Model): integrated both individual- and community-level variables to examine their combined effects. For significance testing, a 95% posterior credible interval was utilized to evaluate posterior mean estimates.

## Assessment of convergence

To ensure the reliability of the analysis results, the chain should reach its stationary distribution. Convergence was assessed by examining whether the Gelman-Rubin statistic equals one [10] and both the Bulk Effective Sample Size and Tail Effective Sample Size were adequate [11]. Additionally, trace plots were inspected for each chain to confirm they exhibit good mixing and examine density plots to assess their smoothness. Furthermore, autocorrelation was assessed to evaluate the independence of samples [10, 12].

## Measures of clustering and heterogeneity

The measures of variation (random effects) were summarized using Intra-Class Correlation (ICC), Median Odds Ratio (MOR), and Proportional Change in Variance (PCV) to assess variability across enumeration areas (clusters). The ICC quantifies the proportion of total variance attributable to differences between clusters, relative to the overall variance. It is computed as the ratio of between-cluster variation to total variation, estimating the extent to which the grouping at the second level contributes to the total variance in the dependent variable. This statistic helps determine whether heterogeneity at the cluster level significantly influences the intercept of individual-level observations[13].

$$ICC=\frac{{(\sigma}^{2}({uo}_{j})}{\sigma^{2}\left( {uo}_{j} \right)+\frac{\pi^{2}}{3}} (5)$$

Where, ${(\sigma}^{2}\left( {uo}_{j} \right)$ is the community (cluster) level variance and $\frac{\pi^{2}}{3}$ is the standard logistic distribution, that is, the assumed household variance component, which is$\frac{\pi^{2}}{3}\approx3.29$.

The Median Odds Ratio (MOR) quantifies the median relative change in the likelihood of an event occurring when comparing identical individuals from two randomly selected clusters, ranked by proportion. In a mixed-effects model, the random effects are assumed to follow a normal distribution, ${uo}_{j} \sim N(0, \sigma^{2})$. The MOR may be calculated as [14]:

$${\mathrm{MOR}=exp}^{\left( \sqrt{2\sigma^{2}}\Phi^{-1}(0.75 \right))} (6)$$

Where, $\Phi^{-1}$ denotes the inverse of the standard normal cumulative distribution function with mean zero and variance one, $\Phi^{-1}(0.75)$ is the 75th percentile, and $exp$is the exponential function. The measure is always greater than or equal to 1. If the MOR is 1, there is no variation between clusters (no second-level variation). If there is considerable between-cluster variation, the MOR will be large.

The Proportional Change in Variance (PCV) measures the overall variation in each model that can be attributed to individual- and/or community-level factors. It is calculated by comparing the variance in a reference model with the variance in a subsequent model, helping to assess how much of the variance is explained by the inclusion of additional predictors [15, 16]:

$$PCV=({\delta_{A}-\delta_{B})}/{\delta_{A}} \left( 7 \right)$$

where $\delta_{A}$ , is the variance of the initial model, and $\delta_{B}$ is the variance of the model with more factors.

# Result

## Bayesian inference

### Model diagnostics and comparison

Four models were compared using LOO-CV criteria for model comparison and selection. Based on this criterion, the Cumulative proportional odds model was found to be the better model having the smallest LOO-CV value (Figure S1).

Figure S1**: Bar plot of LOO-CV values for different Bayesian categorical model**

Before interpreting the cumulative logit model's findings, the validity of the proportional odds assumption was checked. This assumption was not breached, as evidenced by the approximate likelihood-ratio test for the proportionality of odds across response categories yielding a value of 0.386, which exceeds 0.05. Consequently, the cumulative logit model's outcomes can be confidently presented and further analyzed.

After the best model was selected, four models were fitted and compared in examining the individual and community-level factors associate with vaccination status. The four models comprised an empty model (Model I) which shows the variations in the vaccination status in the absence of any explanatory variable, Model II=Individual-level variables, Model III which comprised of Community-level variables and Model IV, the final model comprised for both individual-and community-level variable.

## Assessment of convergence

Model convergence was confirmed with the Gelman-Rubin statistic equal to 1, and the Bulk Effective Sample Size and Tail Effective Sample Size for all parameters were above 2500 and 3100, respectively. Additionally, the trace plots showed good mixing, and the density plots were smooth (Figure S2).

Figure S2**: Density and trace plot of some of the covariates and parameters**

# Reference

1. Andrew, G., B. Carlin John, S. Stern Hal, B. Dunson David, and V. Aki, *Bayesian data analysis.* Chapman: Hall/CRC, 2013.

2. Bürkner, P.-C., *brms: An R package for Bayesian multilevel models using Stan.* Journal of statistical software, 2017. **80**: p. 1-28.

3. Hoffman, M.D. and A. Gelman, *The No-U-Turn sampler: adaptively setting path lengths in Hamiltonian Monte Carlo.* J. Mach. Learn. Res., 2014. **15**(1): p. 1593-1623.

4. Ananth, C.V. and D.G. Kleinbaum, *Regression models for ordinal responses: a review of methods and applications.* International journal of epidemiology, 1997. **26**(6): p. 1323-1333.

5. Bürkner, P.-C. and M. Vuorre, *Ordinal regression models in psychology: A tutorial.* Advances in Methods and Practices in Psychological Science, 2019. **2**(1): p. 77-101.

6. Tutz, G., *Ordinal regression: A review and a taxonomy of models.* Wiley Interdisciplinary Reviews: Computational Statistics, 2022. **14**(2): p. e1545.

7. Liu, X., A.A. O'Connell, and H. Koirala, *Ordinal regression analysis: Predicting mathematics proficiency using the continuation ratio model.* Journal of Modern Applied Statistical Methods, 2011. **10**: p. 513-527.

8. Liu, Q., B.E. Shepherd, C. Li, and F.E. Harrell Jr, *Modeling continuous response variables using ordinal regression.* Statistics in medicine, 2017. **36**(27): p. 4316-4335.

9. Mankelkl, G. and B. Kinfe, *Sociodemographic factors associated with anemia among reproductive age women in Mali; evidenced by Mali malaria indicator survey 2021: spatial and multilevel mixed effect model analysis.* BMC Women's Health, 2023. **23**(1): p. 291.

10. Brooks, S.P. and A. Gelman, *General methods for monitoring convergence of iterative simulations.* Journal of computational and graphical statistics, 1998. **7**(4): p. 434-455.

11. Vehtari, A., A. Gelman, D. Simpson, B. Carpenter, and P.-C. Bürkner, *Rank-normalization, folding, and localization: An improved R ̂ for assessing convergence of MCMC (with discussion).* Bayesian analysis, 2021. **16**(2): p. 667-718.

12. Gelman, A. and D.B. Rubin, *Inference from iterative simulation using multiple sequences.* Statistical science, 1992. **7**(4): p. 457-472.

13. Austin, P.C., H. Stryhn, G. Leckie, and J. Merlo, *Measures of clustering and heterogeneity in multilevel P oisson regression analyses of rates/count data.* Statistics in medicine, 2018. **37**(4): p. 572-589.

14. Rabe-Hesketh, S. and A. Skrondal, *Multilevel and longitudinal modeling using Stata*. 2008: STATA press.

15. Larsen, K. and J. Merlo, *Appropriate assessment of neighborhood effects on individual health: integrating random and fixed effects in multilevel logistic regression.* American journal of epidemiology, 2005. **161**(1): p. 81-88.

16. Austin, P.C., P. Wagner, and J. Merlo, *The median hazard ratio: a useful measure of variance and general contextual effects in multilevel survival analysis.* Statistics in Medicine, 2017. **36**(6): p. 928-938.
